# Dual-Specificity Phosphatases in Immunity and Infection: An Update

**DOI:** 10.3390/ijms20112710

**Published:** 2019-06-02

**Authors:** Roland Lang, Faizal A.M. Raffi

**Affiliations:** Institute of Clinical Microbiology, Immunology and Hygiene, Universitätsklinikum Erlangen, Friedrich-Alexander-Universität Erlangen-Nürnberg, 91054 Erlangen, Germany

**Keywords:** MAPK phosphatase, atypical DUSP, macrophage, T cell, cytokines, inflammation

## Abstract

Kinase activation and phosphorylation cascades are key to initiate immune cell activation in response to recognition of antigen and sensing of microbial danger. However, for balanced and controlled immune responses, the intensity and duration of phospho-signaling has to be regulated. The dual-specificity phosphatase (DUSP) gene family has many members that are differentially expressed in resting and activated immune cells. Here, we review the progress made in the field of DUSP gene function in regulation of the immune system during the last decade. Studies in knockout mice have confirmed the essential functions of several DUSP-MAPK phosphatases (DUSP-MKP) in controlling inflammatory and anti-microbial immune responses and support the concept that individual DUSP-MKP shape and determine the outcome of innate immune responses due to context-dependent expression and selective inhibition of different mitogen-activated protein kinases (MAPK). In addition to the canonical DUSP-MKP, several small-size atypical DUSP proteins regulate immune cells and are therefore also reviewed here. Unexpected and complex findings in DUSP knockout mice pose new questions regarding cell type-specific and redundant functions. Another emerging question concerns the interaction of DUSP-MKP with non-MAPK binding partners and substrate proteins. Finally, the pharmacological targeting of DUSPs is desirable to modulate immune and inflammatory responses.

## 1. Introduction

Reversible phosphorylation plays a fundamental role in signal transduction in physiological and pathological processes. Kinases phosphorylate multiple substrates, including other kinases and transcription factors, allowing the rapid transmission of signaling information to the nucleus and cellular responses at the level of gene expression and post-transcriptional regulation. The kinases and their substrates required for key biological processes, such as cell proliferation, cytokine or antigen receptor signaling, have been intensively studied by biochemical, genetic and pharmacological methods. In fact, several kinase inhibitors have been successfully developed for clinical application in oncology and autoimmune diseases. In contrast, the function and specificity of the approximately 200 phosphatases encoded in the human and murine genomes are much less well characterized, although the dephosphorylation of proteins and other substrates is an equally vital molecular switch to initiate or terminate signaling. One reason for this apparent neglect of phosphatase research has been the notion that the specific pharmacological targeting of phosphatases is not possible. However, the recent development of specific allosteric inhibitors for SHP-1, SHP-2 [1] and several other phosphatases has led to a renewed interest in drug discovery for phosphatases [2,3].

Dual-specificity phosphatases (DUSPs) come in two flavors, first as MAPK phosphatases and second in the form of atypical DUSPs that can have diverse substrates. Given the vastly documented importance of MAPK signaling in innate and adaptive immune cells, it is not surprising that several members of the DUSP-MKP family play essential roles in the regulation of antigen and pattern recognition receptor-driven immune responses. The research into the immunological functions of DUSP-MKP began more than a decade ago with the demonstration of the non-redundant functions of DUSP1/MKP-1, DUSP2/PAC1 and DUSP10/MKP-5 in the immune system (reviewed at the time by us and others [4,5,6]. Since then, the involvement of other DUSP-MKP family members in immune responses to infection and in inflammation has been investigated quite intensely by many groups in the mouse and human system. The phenotypes of DUSP-deficient mice and the mechanistic studies performed established that most members of the DUSP-MKP contribute to immune regulation and, overall, corroborate the notion that regulation of MAPK signaling is the prime mechanism of this group of phosphatases. However, results from the studies performed in the last decade also point to several open questions regarding the spectrum of substrates and molecular interaction partners and the cell type-specific expression and function of DUSP-MKP. Hence, the major aim of this review will be to give an overview of the main findings from the literature on DUSP-MKP in the immune system. For several members of the large group of atypical DUSPs (lacking a specific kinase-interaction motif), evidence is emerging for a function in immunity. Therefore, the state of the literature on DUSP3, DUSP11, DUSP12, DUSP14 and DUSP22 is also reviewed here. We will conclude by highlighting, in our opinion, the most important questions for ongoing research on the role of DUSPs in the immune system.

## 2. DUSP in Immunity and Infection

### 2.1. DUSP Family Phosphatases: A General Overview

**Figure 1:**
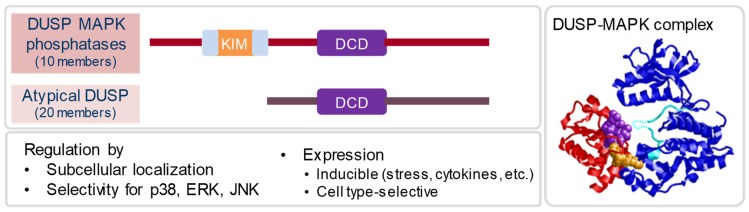
Dual-specificity phosphatases (DUSP): domain structure, mode of action, and levels of regulation. The 10 members of the classical DUSP-MAPK phosphatases contain a MAPK-binding kinase-interaction motif (KIM) conferring selective binding to ERK1/2, p38 or JNK1/2. Upon binding to MAPK, the DUSP catalytic domain (DCD) dephosphorylates the TXY motif in the activation loop (right panel). Atypical DUSPs lack a KIM and can have more diverse substrates, including phosphorylated RNA (DUSP11). Regulated expression between cell types and after stimulation, different compartmentalization of DUSP, and selectivity in binding to MAPK family members confers specificity of DUSP action in signaling.

DUSP proteins belong to the extended family of tyrosine phosphatases (for an overview, see [7]). They have been grouped in class I of the cysteine-based phosphotyrosine phosphatases (PTP) that carry the CxxxxxR signature motif in the active site of the catalytic domain. Several classical PTPs such as PTP1B, SHP1 and CD45 are in subclass I of class I. Subclass II of dual-specificity/VH1-like PTPs is characterized by a HCxxGxxR signature motif and consists of 63 members. These include the DUSP-MKP (10 members), the small-size atypical DUSPs (15 members), other atypical DUSPs (five members), slingshot phosphatases, phosphatases of regenerating liver (PRL), CDC14s (four members) and PTEN-like phosphatases (eight members). All DUSP-MKP, most small-size atypical DUSPs, and several other subclass II members show specific phosphatase activity against phosphorylated Ser, Thr or Tyr. Some atypical DUSPs, PRLs and PTEN-like proteins have non-amino acid substrates, including triose-phosphorylated RNA (DUSP11), phosphatidylinositol phosphates (PRL3, PTEN, DUSP23), or their substrates are yet unknown. In a narrower sense, we consider here as DUSP family members the ten DUSP-MKP and the 20 small-size and atypical DUSPs (Figure 1). 

#### 2.1.1. MAPK Phosphatases

The specificity of the 10 members of this group for distinct MAPK family members is due to the presence of an N-terminal MAPK-binding domain (MKB) containing a kinase-interaction motif (KIM) (Figure 1). While DUSP6/MKP-3 specifically binds to and dephosphorylates extracellular signal-regulated kinases (ERK)1/2, but not p38 and JNK, DUSP10/MKP-5 binds and inactivates p38, but not ERK1/2, whereas DUSP1/MKP-1 can interact and dephosphorylate ERK1/2, p38 and JNK. Of note, substrate preferences for distinct MAPK in vivo are not always predicted by in vitro binding and catalytic activation studies. The KIM contains positively charged arginine residues which are essential for the specific interaction with the MAPK. As excellently reviewed by Caunt and Keyse [8], the structural details of the binding interactions between DUSP-MKPs and their MAPK partners differ and can explain the specific activities for MAPK family members. Importantly, for some DUSP-MKPs (e.g., DUSP1/MKP-1, DUSP4/MKP-2 and DUSP6/MKP-3), the catalytic activity robustly increases as a consequence of a conformational change in the DSP domain, whereas this is not the case for DUSP5, DUSP10/MKP-5 and DUSP16/MKP-7 [8]. 

The DUSP-MKPs have relatively diverse C-terminal domains that are important for the regulation of protein stability and subcellular localization. PEST sequences in DUSP16/MKP-7 promote ubiquitinylation and proteasomal degradation, whereas phosphorylation of the C-terminus by ERK1/2 increases protein stability [9]. Nuclear export signals in the C-terminal domain govern the subcellular compartmentalization of DUSP6, DUSP8 and DUSP9 in the cytoplasm, while the combined presence of NES and nuclear localization signals in DUSP16 can explain its shuttling between the cytoplasm and nucleus [10]. 

#### 2.1.2. Atypical DUSPs

Members of the 20 small-size and atypical DUSPs [7] lack a designated MKB domain and therefore dephosphorylate less well-defined substrates. Many of these DUSP dephosphorylate proteins with pSer, pThr and pTyr residues, and some of them interact with and regulate MAPK family members. However, activity towards other phosphoproteins is more commonly found in this family, and some members dephosphorylate non-protein substrates such as RNA or lipids. Thus, the field of atypical DUSP research is very diverse at the biochemical and functional level. In the context of this review, we will therefore focus on selected atypical DUSPs with a demonstrated role in the regulation of the immune response (DUSP3, DUSP11, DUSP12, DUSP14, and DUSP22).

### 2.2. Models of DUSP Regulation in the Immune System

Innate and adaptive immune cells rely on rapid kinase-driven signaling to translate the recognition of microbial structures (through TLR and other PRR) or the binding of specific antigen (by the B cell and T cell receptors) into massive transcriptional and post-transcriptional responses required for the swift production of cytokines, antimicrobial effector molecules and efficient clonal expansion in case of lymphocytes.

**Figure 2:**
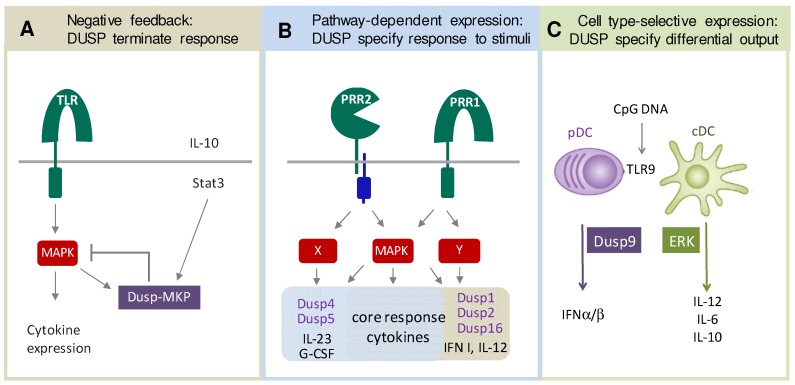
DUSP as regulators and specifiers of innate immune responses. (**A**) Negative feedback regulation of MAPK signaling by activation-induced DUSP upregulation, shown here as example TLR-induced DUSP1 that is enhanced by IL-10-STAT3 signaling. (**B**) Ligands for different pattern recognition receptors trigger overlapping yet distinct gene expression programs, including the differential expression of DUSP family members that then tune the amplitude and kinetics of MAPK activation. (**C**) Activation of the same pattern recognition receptor (here TLR9) in different cell types induces strikingly different cytokine outputs. Differential expression of DUSP genes (here DUSP9 in pDC, but not cDC) contributes to cell type-specificity of signaling and transcriptional responses.

To achieve a balanced immune response, the intensity and duration of these cellular responses has to be controlled and, finally, to be terminated. Several DUSP-MKP and atypical DUSP are strongly upregulated in immune cells in response to the stimulation of PRR or antigen receptors, suggesting that they contribute to this regulatory process through the negative feedback regulation of MAPK and other kinases they interact with. Such negative feedback loops can also receive input from other cues, e.g., cytokines such as IL-10 that enhances expression of DUSP1 (Figure 2A). Prototypic examples of this type of regulation are the inducible nuclear DUSP-MKP DUSP1, DUSP2 and DUSP5, as described in detail below. 

Triggering of different pattern recognition receptors on innate immune cells, such as TLR or C-type lectin receptors, induces overlapping yet distinct transcriptional responses. Ligand- or receptor-specific changes in gene expression are due to differences in signaling pathways and/or their spatio-temporal regulation, and, importantly, control the inflammatory reaction and direct the adaptive immune response. To date, the ligand- or receptor-specific induction of DUSP family genes and their kinetics in macrophages and dendritic cells has not been investigated in detail. However, while the TLR ligands LPS and CpG induce the strong upregulation of DUSP1, DUSP2 and DUSP16 [11], the mycobacterial cord factor TDM caused a marked induction of DUSP4 and DUSP5 [12]. Such ligand- or receptor-related differential expression of DUSP genes may contribute stimulus-specific transcriptional programs through the selective regulation of individual MAPK substrates and the subcellular localization and activity of the individual DUSP (Figure 2B).

The activation of different innate immune cells, e.g., macrophages, conventional or plasmacytoid DC, by the same stimulus can trigger remarkably different cellular outputs. For example, the activation of TLR9 by CpG ODN triggers inflammatory cytokine production in macrophages and cDC but high-level IFNα/β secretion in plasmacytoid DC. While these cell type-specific responses can have many reasons in the spatio-temporal regulation of signaling cascades, the constitutive or inducible expression of different sets of DUSP genes may strongly impact on the specification of the cellular reaction to exactly the same stimulus (Figure 2C). The selective constitutive expression of DUSP9 in plasmacytoid DC is one such example where a specific DUSP is associated with a cell type-specific response [13]. 

### 2.3. Role of Specific DUSP in Immune Responses and Host Defence

#### 2.3.1. DUSP-MKPs

##### DUSP1/MKP-1

This inducible nuclear MAPK phosphatase is expressed at high levels in many cell types in response to serum, growth factors, and environmental stresses (such as high salt and osmotic stress). In cells of the innate immune system, DUSP1 is strongly induced by TLR ligands and further enhanced by concomitant treatment with IL-10 [11]. Dusp1 mRNA is highly unstable, with a t_1/2_ in the range of 15–30 min [11,14]. In addition to regulation at the level of transcription and mRNA stability, DUSP1 levels are controlled by translational regulation, as shown by genome-wide profiling of polysomal mRNAs in resting and LPS-activated macrophages [15]. Similar to other negative feedback inhibitors of signaling, DUSP1 mRNA associated with polysomes was strongly increased after stimulation, indicating derepression of translational efficiency. Post-translationally, DUSP1 protein stability is strongly increased by ERK-mediated phosphorylation [16,17].

In addition to TLR ligands, several other microbial products have been shown to induce DUSP1 expression and to affect MAPK activation states. These include the helminth immunomodulatory cysteine protease inhibitor AvCystatin [18], the pore-forming protein pneumolysin of *Streptococcus (S.). pneumoniae* [19], and the hyphal form of *Candida albicans* [20]. Glucocorticoids are another strong stimulus of DUSP1 expression by macrophages [11,17], mast cells [21] and epithelial cells [22,23]. In fact, the anti-inflammatory effect of glucocorticoids is at least partially mediated by DUSP1, because inhibition of TNF, COX2 and IL-1 expression is resistant to dexamethasone in Dusp1^−/−^ macrophages [24]. A strong phenotype of *Dusp1^−/−^* mice in the LPS challenge model was described by several groups, showing that prolonged and intensified p38 activation in response to TLR4 ligation led to the overshooting production of a subset of LPS target genes including chemokines (CCL3, CCL4), cytokines (TNF, IL-6, IL-10, IL-1), and other inflammatory mediators [25,26,27,28]. In several models of bacterial peritonitis (CLP, *Escherichia coli* injection) and sepsis (*Staphylococcus aureus*), a similar overproduction of cytokines, inflammatory damage to vital organs such as the lung, and increased lethality was found [29,30,31,32]. Lung infection of DUSP1^−/−^ mice with *Chlamydia pneumoniae* led to increased bacterial loads associated with higher levels of IL-6 and chemokines in the lungs [33]. 

**Figure 3:**
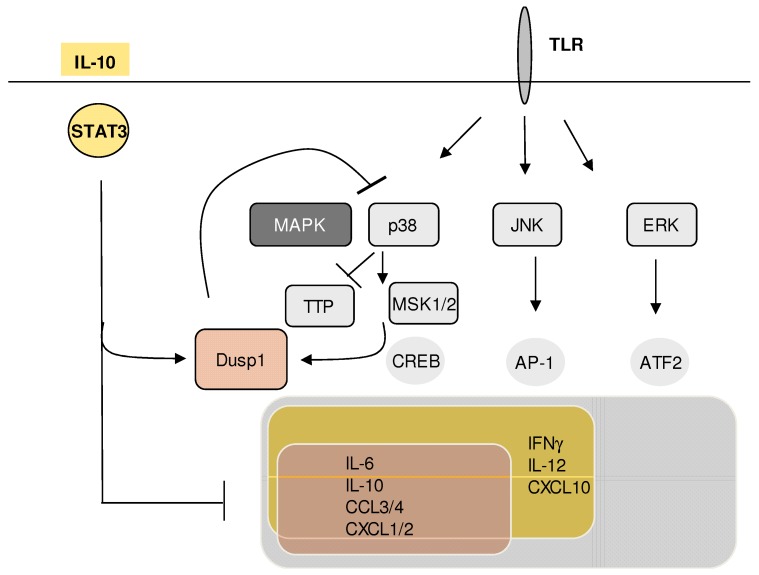
Mechanism of DUSP1 regulation of cytokine expression in Toll-like receptor (TLR)-stimulated macrophages. DUSP1 expression is induced after TLR triggering via MAPK activation and strongly enhanced by IL-10-STAT3 signaling. Preferential inhibition of p38 activity by DUSP1 down-regulates the expression of a subset of cytokines by interfering with MSK1/2-dependent transcription factors and through the control of mRNA decay via the post-translational regulation of TTP. See text for references and details.

These results suggested that the TLR-induced expression of DUSP1 during infection is required to restrain damaging hyper-inflammation, partially mediating the effect of endogenous IL-10. Interestingly, the dramatically increased production of cytokines such as IL-6 and TNF was not efficient in achieving a reduction of pathogen burden. Mechanistically, THE increased activity of p38 in the absence of DUSP1 was shown to enhance inflammatory gene expression through signaling to MSK1/2 and the substrate transcription factors CREB and ATF1 [34] (Figure 3). In addition, the DUSP1-p38-MK2 regulatory module also controls cytokine levels via effects on mRNA stability through THE expression and post-translational modification of the RNA-binding protein TTP: first, TTP mRNA expression is upregulated in the absence of DUSP1; secondly, the phosphorylation of TTP by p38 inactivates its RNA-degrading capacity, thereby increasing the stability of mRNAs for TNF, IL-6, and multiple other inflammatory transcripts [35], including interferon beta [36]. Consequently, unleashed p38 activity in the absence of DUSP1 antagonizes TTP and increases inflammatory gene expression by prolonging stability ([37]; for review, see [38]).

While the phenotype of DUSP1^−/−^ mice in infection and inflammation models was consistent with its role in innate immune cells in vitro, the broad expression in many cell types suggested that it may also be involved in the regulation of adaptive immune responses. Indeed, DUSP1-deficient mice developed pronounced Th17-biased cellular immunity after immunization through an indirect effect of APC-derived IL-6 and IL-1 [39]. In contrast, increased tissue inflammation in the T cell-dependent Experimental Autoimmune Encephalitis (EAE) model of multiple sclerosis was observed in DUSP1-deficient mice and depended on the dysregulated responses of astrocytes and fibroblasts to IL-17 receptor signaling [40]. Given its relatively broad tissue expression in multiple cell types, the clarification of the cell type-specific roles of DUSP1 in the regulation of inflammatory responses will require the use of conditional knockout mice [41,42]. 

##### DUSP2/PAC1

DUSP2 is, like DUSP1, an inducible nuclear protein [43]. It has been cloned from human T cells and is highly induced in lymphocytes after activation [44]. DUSP2 is a downstream target of the tumor suppressor p53 in signaling apoptosis and growth suppression [45]. Dusp2 mRNA was identified as one of the most highly induced transcripts in many activated leukocytes and acts as a positive regulator of inflammatory cell signaling and effector functions [46]. Dusp2^−/−^ mice were protected from inflammation in the ‘K/BxN’ animal model of serum transfer-induced arthritis [46]. Protection was mediated by impairment in the effector responses by mast cells and macrophages that showed decreased phosphorylation of ERK and p38, but increased JNK phosphorylation, and impaired NFAT-AP1 and Elk1 transcriptional activation. Interestingly, JNK inhibition restored ERK activation showing crosstalk between MAPK pathways [46]. 

More recently, DUSP2 was shown to be a negative regulator of STAT3 signaling during the commitment of cells to the Th17 lineage in a T cell transfer model of colitis [47]. Here, the expression of DUSP1, DUSP2 and DUSP4 was upregulated in T cells upon stimulation with anti-CD3 and anti-CD28. Using a co-overexpression system in HEK293T cells, the repression of an IL2-luc reporter by DUSP1, DUSP2 and DUSP4 was found. DUSP2 and DUSP4 synergized in this assay. In this system, the authors also observed the association of DUSP2 with DUSP1 and DUSP4, but not with other co-expressed DUSP proteins. Further, DUSP2 directly catalyzed STAT3 dephosphorylation with physical association between DUSP2 and STAT3 [47]. In mouse models of inflammatory bowel disease (IBD), Dusp2^−/−^ mice developed more severe inflammatory disease, with increased production of Th17 cells via STAT3 signaling. In human IBD, DUSP2 expression was down-regulated by methylation and failed to be induced during T cell activation in PBMCs from patients with ulcerative colitis (UC) [47].

Dusp2 expression was recently reported to be hampered in Batf^−/−^ bone marrow-derived macrophages (BMM) stimulated with the TLR7 ligand R848 [48]. Interestingly, Dusp2 mRNA expression levels in macrophages correlated inversely with the amount of STAT3 phosphorylated on Tyr-705. Although these data are consistent with a dephosphorylation of STAT3 by DUSP2 as reported before [47], Kanemaru et al. did not demonstrate a direct interaction of DUSP2 with STAT3; increased phosphorylation of STAT3 in Batf^−/−^ BMM expressing low levels of Dusp2 may therefore also be an indirect consequence of alterations in cytokine levels (e.g., higher concentrations of IL-10 would cause a similar phenotype). Together, more work is required to validate the unexpected and interesting observations made in Dusp2*^−/−^* mice and regarding its interaction with non-MAPK substrates. The further dissection of the function of DUSP2 would benefit from cell type-specific deletion in mice and further biochemical studies on its interaction with other proteins.

##### DUSP4/MKP-2

Dusp4 cDNA was cloned from PC12 cells and has significant homology with DUSP1/MKP-1 [49], but differs from MKP-1 in tissue distribution. DUSP4/MKP-2 is induced by growth factors, cellular stress, UV-light and by LPS [46,49]. DUSP4 is localized in the nucleus with two putative nuclear localization sequences (NLS1, NLS2) [50] and dephosphorylates selectively ERK and JNK in vitro [51].

DUSP4 deficiency in mice limited the Th1 immune response and increased the susceptibility to infection with the protozoan *Leishmania mexicana* [52]. DUSP4 deficiency in macrophages did not increase the phosphorylation of the previously described substrate ERK, but interestingly increased the LPS-induced phosphorylation of JNK and p38. Macrophages lacking DUSP4 produce increased amounts of the cytokines, IL-6, IL-12 and TNF, and of the prostaglandin PGE_2_. They also express higher levels of the enzyme arginase-1 at baseline and after stimulation with LPS, and decreased levels of inducible nitric oxide synthase (iNOS) in response to LPS or IFNγ, resulting in the strongly reduced production of nitric oxide (NO) [52]. Consistent with the pivotal role for NO in control of *Leishmania* replication, DUSP4-deficient macrophages were unable to control *L. mexicana* parasite infection; however, the capacity for NO production and, in part, to suppress intracellular parasite proliferation could be restored by the arginase-inhibitor nor-NOHA. In vivo, DUSP4^−/−^ mice had a strongly decreased ability to clear parasite growth with a limited Th1 and increased Th2 response [52]. Infection of DUSP4^−/−^ mice with *Toxoplasma gondii*, another intracellular protozoal pathogen, mirrored the increased susceptibility to infection observed in the *L. mexicana* model in terms of increased parasite burden, reduced NO production and increased expression of arginase-1; however, in the case of *T. gondii* infection in Dusp4^−/−^ mice, high arginase-1 levels contribute to parasite control, likely through depletion of L-arginine [53].

Two murine sepsis models revealed that DUSP4 is a positive regulator of inflammation, in contrast to the closely related DUSP1/MKP-1 [54]. Here, Dusp4^−/−^ mice showed improved survival in the high-dose LPS and the cecal ligation and puncture (CLP) model of sepsis, with attenuated levels of systemic IL-1β, IL-6 and TNF. At variance with the findings of an earlier study [52], the phosphorylation of ERK was increased, whereas the phosphorylation of p38 and JNK were decreased in DUSP4-deficient macrophages. Interestingly, DUSP1 induction was increased in the absence of DUSP4 and siRNA knockdown of DUSP1 reversed the cytokine production [54]. As these results indicate an indirect phenotypic effect of the DUSP4 knockout, the combined deletion of both DUSP1 and DUSP4 is desirable to study DUSP redundancy and combinatorial effects during sepsis and inflammation. 

DUSP4 knockout mice were also analyzed in the EAE mouse model of multiple sclerosis. Perhaps not surprisingly, given the phenotype of attenuated inflammation in the previous in vivo models, the severity of clinical disease and of immune cell infiltration in the CNS was attenuated in the absence of DUSP4. Antigen-specific cytokine production by immune cells from spleen and draining lymph nodes was reduced, which could be attributed to a CD4^+^ T cell-intrinsic reduction of proliferation and production of IL-2 and IL-17, as well as to a contribution of impaired upregulation of MHC-II and of costimulatory molecules by DUSP4-deficient DC [55]. 

In CD4^+^ T cells, the deletion of DUSP4 led to increased TCR-induced proliferation and expression of CD25 (the IL-2 receptor alpha chain) [56]. Contrary to expectations, the kinetics and intensity of the T cell receptor-triggered phosphorylation of MAPK were not altered in DUSP4^−/−^ CD4^+^ T cells, nor was the activation of IκB kinase β (IKKβ) affected. These findings indicated that DUSP4 plays a redundant role with regard to the dephosphorylation of its canonical substrates JNK and ERK. Interestingly, DUSP4^−/−^ CD4^+^ T cells responded to IL-2 stimulation with somewhat increased levels of STAT5 phosphorylation, which could explain the hyper-proliferative phenotype [56]. In a HEK 293T cell overexpression system, DUSP4 reduced the phosphorylation of STAT5, and DUSP4 protein could be immune-precipitated with STAT5 from thymocytes [56]. DUSP4 was subsequently shown by the same group to interact with STAT5, requiring both its substrate-interacting and the catalytic domain, which down-regulates STAT5 protein levels [57]. The CD4+ T cell response in the EAE model was characterized by a shift in Th cell differentiation from Th17 to regulatory T cells (Treg) in DUSP4-deficient mice, consistent with a reduced clinical severity in this Th17-dependent inflammation model [57]. Since DUSP4 is highly expressed in Treg, its potential contribution to dampened TCR-induced signaling was analyzed in another study. However, no increase in ERK phosphorylation was found in the absence of DUSP4 [58]. 

DUSP4 also appears to play a role in immunosenescence in human T cells, since it was found expressed at higher levels in the CD4^+^ T cells of elderly donors and associated with weakened expression of the costimulatory molecules CD40L and ICOS-L, as well as the B-cell stimulating cytokines IL-21, IL-4 and IL-17. Importantly, the reduced vaccination-induced T cell-dependent B cell response in the elderly could be restored by silencing DUSP4 expression in CD4^+^ T cells [59]. The defective TCR response in patients with idiopathic CD4 lymphopenia (ICL) has been reported to be due to the increased expression of DUSP4 [60]. ICL is a rare disorder characterized by very low numbers of T cells similar to T cell lymphopenia found in elderly people [61]. Gene-expression profiles in CD4^+^ T cells from patients with ICL compared to healthy donors revealed a strong overexpression of DUSP4 in ICL patients. The repeated stimulation of T cells with anti-CD3 Abs in the ICL patients caused a senescent profile with gradual increase in DUSP4 expression and decreased ERK phosphorylation. However, the siRNA-mediated silencing of DUSP4 restored ERK activation and improved T cell activity. 

Together, DUSP4 has important functions in both innate and adaptive immune cells, which appear to be only in part due to the regulation of its classical substrates JNK and ERK MAPK. Whereas in human T cells, the effects of DUSP4 overexpression and silencing could be explained by its control of ERK activation levels, in murine CD4^+^ T cells, the mechanism of DUSP4 regulation of proliferation and Th differentiation appears to act at the level of STAT5 activation and stability. This mechanism may involve the direct binding and dephosphorylation of STAT5 by DUSP4 [56,57]. 

##### DUSP5/hVH-3

DUSP5 is a nuclear MKP and inducible by heat shock and growth factors in mammalian cells [62]. DUSP5 binds directly to and inactivates ERK MAPKs, but not other MAPK family members [63]. Its nuclear localization leads to the anchoring of ERK in the nucleus [63] and strict compartmentalization of ERK de-phosphorylation [64]. Paradoxically, in DUSP5^−/−^ cells, a decrease in sustained cytoplasmic ERK phosphorylation was observed after serum stimulation, that could be explained by a relieving effect of DUSP5 on the ERK-mediated inhibition of upstream RAF kinases [65].

Computational modeling and molecular dynamics analysis of the binding of DUSP5 to phosphorylated ERK2 suggests that the N-terminal ERK-binding domain of DUSP5 first contacts the C-terminal lobe of dual phosphorylated ERK, followed by arranging the linear DUSP5 linker on the ERK2 groove, which brings the catalytic DUSP5 domain in close proximity to the phosphorylated tyrosine and threonine residues of ERK2 [66]. The DUSP5 linker appears to play a critical role in the specific binding to ERK, explaining substrate specificity for ERK [66]. 

The inducible expression of DUSP5 in the immune system was first identified in T cells after stimulation with cytokines signaling through the common gamma chain as negative feedback regulator of ERK activation [67]. During T cell development in the thymus, DUSP5 expression is increased during the transition from double-positive thymocytes to single-positive CD4^+^ T cells [68]. The transgenic overexpression of DUSP5 blocked T cell development at the double-positive stage, whereas in mature CD4^+^ T cells, it impaired the proliferative response to IL-2 stimulation [69]. Deletion of the Dusp5 gene did not cause an obvious phenotype in the murine immune system, but affected memory/effector CD8^+^ T cell populations after viral infection [70]. DUSP5^−/−^ T cells proliferated more strongly and underwent more cell death [70]. DUSP5 is a target of the DNA-damage induced transcription factor p53; whether a negative feedback loop between DUSP5 and p53 activity is operative in T cells remains to be established. 

Eosinophilic granulocytes are induced by helminth infection and during the resolution phase of inflammatory responses. They express considerable levels of DUSP5 under basal conditions (www.immgen.org) and strongly upregulate DUSP5 mRNA and protein levels following stimulation with IL-33 [71]. Increased numbers of eosinophils were observed in bone marrow, blood and spleen of *Nippostrongylus brasiliensis*-infected DUSP5^−/−^ mice compared to wild-type mice, with a decrease in apoptotic cells. Microarray analysis of IL-33-treated DUSP5^−/−^ eosinophils revealed the increased expression of CD69 and Spred2, genes essential for eosinophil activation. DUSP5^−/−^ eosinophils had increased ERK activation after IL-33 treatment, which enhanced the expression of anti-apoptotic Bcl-XL and cell survival [71]. 

In macrophages, DUSP5 expression was induced by the LPS stimulation of TLR4 and reduced cytokine production in RAW264.7 macrophages at the level of ERK1/2-dependent AP1 activation [72]. The stimulation of macrophages with the mycobacterial cord factor trehalose-6,6-dimycolate also upregulated DUSP5 expression, independent of the cord factor receptor Mincle (9). ERK1/2 signaling plays an important role in M-CSF-driven macrophage proliferation and differentiation. DUSP5 is upregulated by M-CSF in myeloid cells and acts as a negative feedback regulator of ERK1/2 activation. Its overexpression increased M-CSF driven proliferation and blocked macrophage differentiation [73]. Instead, DUSP5 directed progenitor cells to differentiate towards granulocytes in response to M-CSF [73], which is in line with data showing that the inhibition of ERK1/2 favors granulocyte over monocyte development [74].

Since DUSP5 is expressed and regulated in many innate and adaptive immune cell types, in vivo analysis of its function in specific immune responses would be aided by conditional knockout mice, that have not been reported yet. 

##### DUSP6/MKP-3

DUSP6 is a cytoplasmic MAPK phosphatase with high selectivity for ERK1/2 due to high affinity binding of the DUSP6 N-terminal KIM to ERK1/2 and a binding-induced conformational change activating the catalytic domain [75]. DUSP6 is constitutively expressed in several immune cell types, including CD4^+^ T cells [76], dendritic cells [13], macrophages [11] and microglia [77]. DUSP6^−/−^ mice have increased ERK phosphorylation in the heart, spleen, kidney, brain and fibroblasts, but are otherwise healthy and fertile [78]. 

DUSP6^−/−^ CD4^+^ T cells responded to TCR stimulation with stronger ERK1/2 phosphorylation, proliferation and IFNγ production, but on the other hand made less IL-17, showed increased apoptosis and a reduced Treg function. In the IL-10 knockout mouse model of spontaneous colitis, additional DUSP6 deficiency exacerbated disease symptoms, indicating a regulatory function of DUSP6, which was confirmed by increased IFNγ production from colonic and mesenteric lymph node CD4^+^ T cells [79]. DUSP6 was required for activation-induced glycolysis in CD4^+^ T cells and for IL-21 production by follicular T helper cells (Tfh) [80]. DUSP6 expression in human CD4^+^ T cells increases with age, due to declining levels of miR-181a, which reduces the sensitivity of TCR-triggering and results in suboptimal expression of activation markers and reduced proliferation [81]. The allosteric DUSP6 inhibitor BCI restores full responsiveness to TCR stimulation, suggesting that DUSP6 could be a promising target for improving cellular immune responses to vaccination in the elderly [81].

In macrophages, DUSP6 is also constitutively expressed and attenuates ERK phosphorylation. Hyperoxia inactivates DUSP6 catalytic activity in macrophages, leading to increased ERK function and the induction of a pro-survival expression program [82]. The pharmacological inhibition of DUSP6 in macrophages by BCI led to attenuated cytokine production through Nrf2-signaling and decreased NFκB activation [83]. In macrophages infected with *Leishmania major*, triggering of the activating receptor CD40 reciprocally regulates DUSP1 and DUSP6, and overexpression of DUSP6 promotes control of leishmanial replication [84]. Thus, depending on the cell type (CD4*^+^* T cells versus macrophages), interfering with DUSP6 activity appears to result in immune restoration or the impairment of innate cytokine production.

##### DUSP9/MKP-4

DUSP9 is an ERK1/2-selective cytoplasmic MKP with an essential role in placental function during gestation [85,86]. Tetraploid rescue experiments showed that despite high-level DUSP9 expression in tubular epithelial cells in the ascending loop of Henle, kidney function in the absence of DUSP9 is normal [85]. DUSP9 is highly expressed in embryonic stem cells in a BMP4-dependent manner and appears to contribute to stemness [87]. Polymorphisms in the human DUSP9 gene were repeatedly associated with increased risk for diabetes in genome-wide association studies (GWAS) [88,89], consistent with a protective function of DUSP9 in the development of insulin resistance in a transgenic mouse model [90]. 

In the immune system, DUSP9 expression is weak in most cells types, but constitutively strong at the mRNA and protein level in murine plasmacytoid DC (pDC) [13]. This cell type is specialized in producing high-level type I IFN in response to the stimulation of TLR7/9 by nucleic acids, which has been attributed to constitutive high IRF7 levels, but is mechanistically incompletely understood. Compared to conventional DC (cDC), triggering TLR9 in pDC induces very little ERK1/2 activation, correlating with high DUSP9 levels. In addition, retrovirally enforced DUSP9 expression in cDC attenuated MAPK activation and increased IFNβ expression, showing that DUSP9 shapes cytokine/interferon production in DC by controlling ERK1/2 activation [13]. However, the conditional deletion of DUSP9 in CD11c-expressing DC did not restore ERK1/2 activation in pDC and only weakly reduced IFNβ and IL-12 expression in response to TLR9 stimulation [13]. These results indicate that either the lack of ERK activation in pDC is caused by intrinsic differences in signaling between pDC and cDC, or, alternatively, other phosphatases compensate for the absence of DUSP9, which may include DUSP5, DUSP6 or other DUSP family members expressed in pDC. Testing these hypotheses will require the simultaneous deletion of multiple DUSP genes in pDC, which is difficult to achieve by breeding conditional knockout mouse lines and may be facilitated by CRISPR/Cas9 technology. 

##### DUSP10/MKP-5

DUSP10 is the only DUSP protein carrying an extended N-terminal domain of unknown function. DUSP10 can be found in the cytoplasm and in the nucleus, and shows selective phosphatase activity towards JNK and p38 MAPK [91,92]. 

DUSP10 was already defined in 2004 by Dong and coworkers as a regulator of the innate and adaptive immune system [93]. In T cells, DUSP10 down-regulates TCR-induced IFNγ-production [93], which was also observed in a murine malaria model during infection of DUSP10^−/−^ mice with *Plasmodium yoelii* [94]. In this case, the enhanced killing of parasites and survival correlated with increased IFNγ production by T cells, which was attributable to enhanced stimulatory capacity of Dusp10^−/−^ splenic DC [94]. DUSP10 plays a host-protective role in LPS-induced vascular injury by limiting ROS production [95] and in sepsis-induced lung injury [96]. On the other hand, DUSP10 limits production of type I IFN by macrophages infected with influenza virus, leading to a more rigorous IFN response to influenza infection in DUSP10 ^−/−^ mice, better control of viral replication and increased survival [97]. While MAPK phosphorylation was largely unaltered in DUSP10^−/−^ cells following stimulation with the virus, the high-level induction of IFN I was associated with the increased phosphorylation of IRF-3, suggesting that IRF-3 may be a non-MAPK substrate of DUSP10 [97]. Similarly, in an earlier study, an interaction of DUSP10 (and DUSP1) with IRF-3 was shown by proximity ligation assay (PLA) in macrophages stimulated with a combination of TLR4 and TLR9 ligands (LPS and B-DNA), or infected with *Listeria monocytogenes* [98]. Since high-level type I IFN has a detrimental role in listeriosis, the inhibition of IRF-3 activation by TLR-induced DUSP10 or DUSP1 may have a beneficial role in control of bacterial replication [98]. 

Manley et al. have analyzed changes in DUSP gene expression in primary bronchial epithelial cells in response to rhinovirus infection and observed that DUSP10 was transiently increased followed by down-regulation after 8 h [99]. Functionally, DUSP10 attenuated chemokine expression in response to virus-induced IL-1β production, as demonstrated by siRNA knockdown of DUSP10 [99]. 

DUSP10 has recently been shown to constrain IL-33-mediated cytokine production by memory-type pathogenic Th2 cells [100]. The expression of DUSP10 was high in these Th2 cells but low in ILC2, which responded to IL-33 with much stronger induction of IL-5 and IL-13. This differential expression was not observed for other DUSP family members and was accompanied by an attenuated activation of p38 MAPK in Th2 cells compared to ILC2. Importantly, the CRISPR/Cas9-mediated deletion of functional DUSP10 in Th2 cells increased both p38 phosphorylation and IL-5/IL-13 production after stimulation with IL-33, whereas the overexpression of DUSP10, but not its phosphatase-dead mutant, inhibited IL-5 and IL-13 in ILC2 cells [100]. 

##### DUSP16/MKP-7

DUSP16 was identified as an LPS-inducible MKP in RAW264.7 macrophages with selectivity for JNK already in 2001 [10,101,102]. DUSP16 possesses a long C-terminal domain containing sequence motifs that control the protein’s stability (PEST sequence) and localization (NES and NLS). In addition, the phosphorylation of DUSP16 at Ser446 increases the half-life of the protein [9]. DUSP16 can shuttle between cytoplasm and nucleus [10] and may therefore inhibit JNK in a compartmentalized fashion. DUSP16 also can bind to activated ERK in the cytoplasm and thereby interfere with nuclear ERK activation [103]. In addition to the full-length DUSP16 isoform A1, an alternative transcript encoding isoform B1 is generated by alternative splicing at comparable levels of steady state mRNA [104]. Due to the lack of high-quality antibodies for the detection of endogenous levels of DUSP16, the relative protein levels of both isoforms in different cell types have not been determined. Overexpressed DUSP16 A1 and B1 are readily detected in 293T cells, indicating that both are reasonably stable. 

The interaction of the N-terminal domain of DUSP16 with the MAPK JNK1 has recently been analyzed in great detail by crystal structures and biochemical assays [105]. Interestingly, JNK1 binds to the 285FNFL288 segment of the DUSP16 catalytic domain (which is absent in the B1 isoform). This interaction was also critical for the dephosphorylation of JNK1 by DUSP16 in vitro and for the anti-apoptotic activity of DUSP16 after UV irradiation [105]. In contrast, the D-motif (or KIM) in the MKB-domain of DUSP16 was not required for binding to JNK1. While this work revealed an important function for the FXF motif, that is conserved in other DUSP, for the binding and regulation of JNK1, the D-motif (=KIM) appears to control the binding of DUSP16 and of DUSP10 to p38 [106]. 

**Figure 4:**
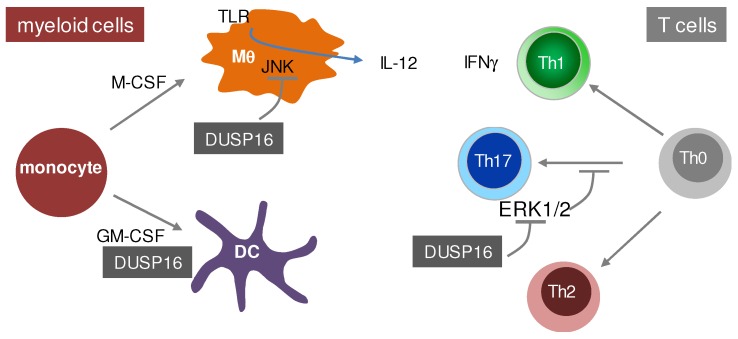
Role of DUSP16 in innate and adaptive immune cells. Analysis of CD4^+^ T cell compartment and myeloid cells derived from radiation chimeras with a DUSP16-deficient hematopoietic system was performed by two labs [104,107].

Two labs have made use of an ES cell line with an insertion of a β-Galactosidase/Neomycin gene-trap in the fourth intron of the DUSP16 gene to generate DUSP16-deficient mice. Consistently, these mice died in the perinatal period for an as yet unknown reason [104,107]. Recently, it was demonstrated that DUSP16 deficiency in these gene-trap mice strongly enhances cell death in sensory neurons upon NGF withdrawal in vitro and a loss of sensory innervations in DUSP16-deficient embryos in vivo [108]. Moreover, DUSP16-deficient embryos develop hydrocephalus due to expansion of neural progenitors and blockade of cerebrospinal fluid circulation through the midbrain aqueduct [109]. The generation of radiation chimeras showed a largely normal reconstitution of the hematopoietic system by DUSP16-deficient homozygous gene-trap fetal liver cells and allowed the characterization of the role of DUSP16 in immune cells. DUSP16-deficient CD4^+^ T cells have a cell-intrinsic defect in Th17 polarization, whereas IFNγ-producing Th1 cells and IL-4-producing Th2 cells are not affected [107]. This effect is dependent on increased ERK1/2 MAPK activation in DUSP16-deficient T cells following TCR triggering, as the MEK1-inhibitor U0126 restored Th17 differentiation [107]. Analysis of innate immune activation by the TLR4 ligand LPS showed a largely comparable cytokine and chemokine output as in WT mice, with the exception of IL-12p40 which was overproduced in DUSP16-deficient mice in vivo, an effect that could be attributed to macrophages, but not DC, in vitro and was dependent on JNK activity [104]. Together, the data in the DUSP16 gene-trap mice indicate important cell type-specific roles of this MKP in the proliferation, differentiation and cytokine response of macrophages, DC and T cells (Figure 4). However, the perinatal lethality of the DUSP16 gene-trap mice and the need to generate radiation chimeras impede the straightforward experimental analysis. In addition, the expression and function of DUSP16 in multiple immune cell types can result in complex phenotypes. Therefore, the generation of conditional DUSP16 knockout mice is needed to overcome these obstacles and to investigate the cell type-specific functions of DUSP16 in detail.

#### 2.3.2. Atypical DUSP with an Emerging Function in the Immune System

##### DUSP3/VHR

The *DUSP3* gene was cloned in 1992; DUSP3 is also known as Vaccinia-H1-related phosphatase (VHR) and is one of the smallest known phosphatases (21 kDa) [110]. The structure, function and regulation of DUSP3 have recently been reviewed [111]. VHR has been reported to be constitutively expressed in T cells, where it dephosphorylates ERK and JNK, but not p38 [112]. As atypical DUSP, DUSP3/VHR lacks a MAPK-binding motif. Still, the reported substrates of DUSP3 include the MAPK family members ERK1/2 [112], p38 [113] and JNK [114]. Non-MAPK substrates of DUSP3 include STAT5 (see below) and several nucleolar proteins involved in the DNA damage response and in DNA/RNA regulation [115]. 

*DUSP3* mRNA expression levels were associated with resistance or susceptibility in infectious disease both in murine models and in humans. First, the down-regulated expression of *DUSP3* was linked to susceptibility to *Staphylococcus aureus* sepsis in A/J mice and in humans with *S. aureus* bloodstream infection [116]. Knockdown of DUSP3 in mouse macrophages led to increased cytokine production in response to *S. aureus* through the enhancement of NFκB activation [116]. Second, *DUSP3* mRNA was identified as part of a transcriptional signature for the diagnosis of tuberculosis from human peripheral blood in two independent studies [117,118]. Remarkably, *DUSP3* belonged to a set of three genes (with *GBP5* and *KLF2*) that were diagnostic for active tuberculosis in eight independent datasets from ten countries [119]. This diagnostic correlation of *DUSP3* expression in peripheral blood cells with active tuberculosis has not been linked to a function of this phosphatase in the host yet. 

DUSP3 can inhibit IFNβ-induced STAT5 phosphorylation on Tyr-694/699 when overexpressed in HEK293T cells [120]. Similar to its function as a MAPK phosphatase for ERK/JNK, the dephosphorylation of Stat5 by DUSP3 required its phosphorylation at position Tyr-138 [120]. The binding specificity of DUSP3 for STAT5 has been investigated in a bioinformatics approach combining molecular modeling, docking and molecular dynamics simulations, which revealed a binding interface at the catalytic domain of DUSP3 that interacts with the SH2-domain of STAT5 and supported a model where DUSP3 binding at one SH2-domain facilitates the release of the flexible STAT5 C-terminus as a prerequisite for its dephosphorylation [121]. 

Loss of DUSP3 has been shown to be associated with cell-cycle arrest and senescence. The expression of *DUSP3* was undetected in G1 and was gradually increased during the progression of HeLa cells to S phase [122]. *DUSP3^−/−^* mice are fertile and healthy. However, DUSP3 deficiency led to a decrease in neovascularization and angiogenesis, likely due its expression in endothelial cells [123].

DUSP3 was shown by Rahmouni and colleagues to be a non-redundant regulator of the innate immune response to endotoxin or in polymicrobial infection [124,125]. The expression of *DUSP3* was higher in macrophages and monocytes than neutrophils, B cells and T cells. The authors elucidated that protection of DUSP3-deficient mice from septic shock involves polarization to M2-like macrophages and the decreased production of TNF. Bone marrow transfer of *DUSP3^−/−^* to irradiated WT mice showed LPS resistance in the recipients, demonstrating that the phenotype is indeed due to the deletion of *DUSP3* in hematopoietic cells, especially in monocytes [124]. Mechanistically, DUSP3-deficient macrophages unexpectedly showed reduced LPS-triggered ERK1/2 phosphorylation, whereas p38 and JNK activation kinetics were unaltered [124]. In a follow-up study, the protection against sepsis in DUSP3-deficient mice was revealed to be sex-specific for female mice and to involve estrogen-dependent reduction in ERK phosphorylation and enhanced M2 macrophage polarization [125]. The same group investigated the function of DUSP3 in tumor-associated macrophages in a Lewis Lung carcinoma (LLC) metastasis model, where *DUSP3^−/−^* macrophages displayed enhanced recruitment to LLC-bearing lungs and promoted metastatic tumor growth [126]. 

DUSP3 is highly expressed in human and mouse platelets. *DUSP3^−/−^* platelets displayed reduced activation and aggregate formation in response to collagen exposure in vitro, and DUSP3-deficient mice showed impaired pulmonary thromboembolism in vivo [127]. Mechanistically, the phosphorylation of the kinase SYK induced by platelet stimulation with collagen-related peptide (binding to Glycoprotein VI) or by the CLEC2-agonist Rhodocytin was selectively and strongly reduced in the absence of DUSP3. In fact, DUSP3 deficiency phenocopies the knockout of CLEC2 and GPVI [128], suggesting the functional relevance of SYK-regulation by DUSP3. It remains to be elucidated how DUSP3 participates in the activation of SYK. No hyperphosphorylation of tyrosine residues was detected by immunoblot. On the other hand, selective inhibitors of DUSP3 phosphatase activity replicated the impaired activation of SYK and platelet aggregation, indicating that DUSP3 may act by dephosphorylating phosphothreonine or serine residues on proteins in the CLEC2 or GPVI signaling complex [127]. 

In addition to the compounds identified and used in the platelet study by Rahmouni and colleagues, several groups have reported the development of DUSP3 inhibitors [129,130]. While these compounds have not been used in vivo yet, their availability demonstrates that the drug-targeting of DUSP3 is feasible. The identification of yet unknown substrates of this atypical DUSP should greatly facilitate the elucidation of the biological responses and signaling pathways it regulates. 

##### DUSP11

This atypical DUSP is to date unique in its substrate specificity for phosphorylated RNA. Already in 1998 Yuan et al. identified DUSP11/PIR (for Phosphatase that interacts with RNP-complex-1) as a nuclear protein with nuclear RNA-ribonucleoprotein complexes [131]. Subsequently, DUSP11/PIR was shown to remove two phosphate groups from 5′-triphosphorylated RNA, with a selectivity of binding to phosphorylated RNA that was orders of magnitude higher over binding to phosphoprotein substrates [132]. 5′-PPP-RNA is characteristic for viral RNAs, including pre-miRNAs of several retroviruses, generated by RNA polymerase III. For proper binding of the 5p-miRNA to the RISC complex, the removal of the 5′-triphosphates is required. Burke et al. could show that the levels of BLV and AdV 5p miRNA were strongly reduced in human and sheep *DUSP11*-null cells, due to the lack of DUSP11 phosphatase activity, leading to inefficient loading onto the Argonaute proteins of the RISC complex [133,134]. These data raised the question whether DUSP11 is specific for viral RNA or also modifies endogenous cellular RNA. No consistent difference in expression of protein-coding RNA was observed when DUSP11 was deleted in two different parental cell lines. However, several RNAP-III-dependent non-coding RNAs of <500 nt length, including Alu-derived elements, were increased in samples treated with Terminator to digest 5′ monophosphorylated RNAs, indicating that DUSP11 affects phosphorylation status and steady state levels of diverse RNAP-III small nc RNA transcripts [134].

If DUSP11 regulates the level of 5′-triphosphorylated endogenous and viral RNAs, their accumulation in the absence of DUSP11 may trigger activation of pattern recognition receptors for viral RNA, especially of RIG-I that binds to 5′-triphosphorylated RNA. A recent study demonstrates that such a mechanism is indeed operating in cells during infection with the Kaposi sarcoma virus (KSV), a herpes virus [135]. Lytic infection with KSV substantially down-regulated the mRNA and protein levels of DUSP11, which was accompanied by an increase in triphosphorylated cellular non-coding vault RNA and RIG-I activity [135]. Thus, the reduced activity of DUSP11 during KSV infection results in host RNA recognition by RIG-I, which results in enhanced antiviral interferon gene expression. 

A more direct anti-viral activity of DUSP11 was found toward the hepatitis C virus (HCV) [136]. Cellular XRN exonucleases can attack HCV RNA, provided that the viral transcripts are not protected by 5′-triphosphorylation. Using different approaches to delete DUSP11 from human cells, Kincaid et al. showed that dephosphorylation by DUSP11 is required to antagonize HCV replication by enabling access of XRN exonucleases to 5′ monophosphorylated HCV RNA [136]. The DNA damage response to irradiation is strongly dependent on the transcription factor p53. Caprara et al. recently identified Dusp11 as an irradiation-induced target gene of p53, which was shown to directly regulate DUSP11 expression by binding to its promoter [137]. 

In a genetic screening approach for phosphatases involved in the control of the replication of *Salmonella typhimurium* in a human cell line, DUSP11 (and Dusp27) were identified as candidates required for efficient inhibition of replication [138]. It is currently unclear, by which mechanism DUSP11 operates in this bacterial infection, and whether intracellular bacteria affect DUSP11 RNA expression similar to what was reported for the herpes virus KSV.

##### DUSP12 (=hYVH1)

This small atypical DUSP protein was initially identified in yeast [139] and is conserved across many species [140]. DUSP12 expression affects DNA content [141] and prevented cell death in mammalian cell lines [142], whereas it is involved in ribosome biosynthesis in yeast [143]. In a recent interactome analysis in human osteosarcoma cells, DUSP12 association with multiple ribonucleoproteins of the 60S and the 40S ribosomal subunits was found [144]. In addition, DUSP12 also bound to the stress granule proteins FMRP and YB1, and functionally contributed to the disassembly of stress granules [144].

Although DUSP12 lacks a MKB-domain, it bound to all three MAPK family members when overexpressed in HEK293T cells [145]. Upon overexpression in RAW264.7 macrophage cells, DUSP12 inhibited LPS-induced p38 and JNK phosphorylation, AP1-dependent promoter activation, and the expression of proinflammatory cytokines (TNF, IL-1, IL-6), whereas IL-10 production was increased [145]. When infected with *Mycobacterium bovis* BCG or *Listeria monocytogenes*, DUSP12-expressing macrophages showed less activation of p38 MAPK, a similar reduction in proinflammatory cytokine levels as observed after LPS, and increased bacterial loads [145]. The scaffold protein STAP-2 binds to both DUSP12 and p38 via its pleckstrin homology domain, which likely is required for the activity of DUSP12 towards p38 in macrophages [145]. To date, no loss-of-function experiments have been reported for DUSP12 in murine or human macrophages. Very recently, the generation of conditional DUSP12 knockout mice by CRISPR/Cas9 has been reported with liver-specific inactivation [146]. These transgenic mice will be an important resource to investigate the function of this broadly expression atypical DUSP in different immune cells during infection and inflammation.

##### DUSP14/MKP6

DUSP14 was identified in 2001 as a CD28-interacting protein in human T cells [147]. Its expression is strongly induced by the CD3/CD28 stimulation of T cells, suggesting it may function as a negative feedback regulator of TCR signaling. Indeed, a dominant negative DUSP14 mutant protein with a cysteine to serine mutation at position 111 in the active site (C111S) induced overshooting JNK and ERK activation after CD3/CD28 stimulation [147]. The phenotype of DUSP14-deficient mice confirmed the notion that this atypical small-size DUSP is a regulator of T cell activation: while the development of lymphoid cells in thymus and spleen was unaltered, a strong hyper-proliferation of DUSP14-deficient T cells in response to CD3 stimulation was observed [148]. Mechanistically, DUSP14 associated with TAB1 after anti-CD3 stimulation, leading to the dephosphorylation and inactivation of TAB1. Consequently, in DUSP14-deficient T cells, the activation of TAK1 and IKK was strongly enhanced, as well as that of the MAPK ERK and JNK [148]. More recently, the same group showed that the phosphatase activity of DUSP14 is dependent on TRAF2-mediated Lys63-ubiquitinylation [149]. Regulation of the NFκB pathway by DUSP14 was also described by another group in HEK293T and HeLa cells, although in this setting, DUSP14 interacted with TAK1 and regulated its phosphorylation, but not with TAB1, in response to IL-1 and TNF [150]. These differences in the molecular binding partners of DUSP14 may reflect species differences. In mice, DUSP14 is required to restrain T cell responses in vivo, since immunization with the model antigen KLH induced enhanced T cell responses and EAE induction with MOG peptide in Complete Freund’s Adjuvant (CFA) induced more severe clinical disease in the mice and overshooting Th1 and Th17 cytokine production [148].

In the context of human infectious diseases, DUSP14 was identified in a large genome-wide expression quantitative trait loci (eQTL) analysis of human DC before and after infection with *M. tuberculosis* and linked to genetic susceptibility to tuberculosis by integration with genome-wide association studies (GWAS) [151]. A more recent study on the role of the genetic polymorphism in DUSP14 found that a low-expression genotype of DUSP14 was accompanied by high transcript levels of IFNGR2 and STAT1 and may thus protect against early TB development [152].

##### DUSP22/JKAP

This atypical DUSP is also known as MKP-X, VHX, JSP1 and LMW-DSP2. The first description of DUSP22 was by Aoyama et al. who cloned it as LMW-DSP2 from mouse testis cDNA [153]. DUSP22 was then identified as JNK-associated phosphatase (JKAP) that is required for full JNK activation in response to TNF [154]. The interaction of DUSP22 with JNK was indirect and the phosphatase activity of DUSP22 was required for JNK activation [154]. In contrast, more recent work reported that DUSP22 acts as a scaffold protein in the ASK1–MKK7–JNK pathway and that its phosphatase activity is not needed for JNK activation [155]. DUSP22 can also bind to STAT3 and inhibit IL-6-induced responses in cell lines (HEK 293T cells) [156]. The expression of DUSP22 is found in many tissues and cell types, with myeloid cells showing higher mRNA expression among immune cells (see ImmGen database). At the protein level, the highest DUSP22 levels were reported in heart and muscle [157]. Over the years, studies in immune cells have provided accumulating evidence for a function of DUSP22 in T cells, in which DUSP22 inactivates the SRC-kinase LCK and thereby inhibits TCR signaling [158]. As a consequence of enhanced TCR signaling, increased T cell proliferation and cytokine production, DUSP22-deficient mice are more susceptible in models of autoimmune disease such as EAE and develop spontaneous inflammation and autoimmunity in old age [158]. In human patients with inflammatory bowel disease (IBD), DUSP22 expression was inversely correlated with disease activity and with the levels of IL-17 and TNF in the inflamed mucosa [159]. The lentiviral overexpression of DUSP22 suppressed CD4^+^ T cell activation status and proliferation, whereas the lentiviral siRNA knockdown of DUSP22 increased Th1/Th17 differentiation [159]. Since the down-regulation of DUSP22 expression in CD4+ T cells was also correlated with the presence and activity of Systemic Lupus Erythematodes (SLE) in human patients, DUSP22 is emerging as a biomarker for several autoimmune diseases [160]. In addition, the reduced expression of DUSP22 has been associated with T cell lymphoma subtypes, where silenced DUSP22 expression and hypomethylation of the DUSP22 promoter were found [161,162]. Interestingly, DUSP22-hypomethylated anaplastic large cell lymphomas with DUSP22 rearrangements are characterized by better prognosis which appears to be linked to the higher expression of costimulatory molecules and reduced PD1/PD1-L activity, and hence stronger immunogenicity [161]. 

## 3. Conclusions and Open Questions

In the last decade, the investigations of DUSP gene expression and function in different cells of the immune system and during immune responses in mouse models and in human disease have greatly expanded our knowledge about the role of this phosphatase family in immunity and inflammation. It is now also well established that in addition to the classical DUSP-MKP, several atypical DUSPs make important contributions to the signaling processes that control innate and adaptive immune cell functions. The field has benefitted tremendously from the generation of knockout mice for DUSP family members, enabling researchers to scrutinize the relevance of individual DUSP genes in immune responses and inflammation in vivo. Much has been learned about the amazing complexity of how the expression and function of different DUSP family members are regulated at the level of transcription, mRNA stability and post-translational modifications. In turn, the investigations into the mechanisms of the DUSP-mediated control of signaling processes have revealed a much more detailed and multi-faceted picture than the simple model of MAPK-binding and dephosphorylation. Elegant studies have (1) shown the importance of the compartmentalization of DUSP activity [65,163], (2) defined the molecular details of DUSP interaction with specific substrates [66,105], and (3) suggested the emerging role of non-MAPK substrates and binding partners of several DUSP [47]. Moreover, the potential of targeting DUSPs by pharmacological means has been demonstrated in several examples identifying specific inhibitors.

Yet, despite this progress, and in some cases because of newly made unexpected findings, there are several open questions and hurdles the DUSP field is facing. These need to be overcome to fully understand the role of the DUSP family in immune cell development, activation and regulation, and to potentially employ this knowledge for the manipulation of DUSP functions in infection, pathological inflammation or vaccination-triggered immunity:

- Obtain better genetic tools to investigate cell type-specific and redundant functions of DUSP family members
  While the power of knockout mouse models has been instrumental to define an immunological role for many DUSP, the expression of several DUSP in diverse immune and non-hematopoietic cell types can produce complex phenotypes and impedes the clear definition of the contribution made by DUSP deficiency in a specific cell type. To date, conditional knockout models have been published for *DUSP1*, *DUSP9*, and *DUSP12*. The generation and wide availability of additional conditional knockout lines is eagerly awaited for more family members.
  In cases where the genetic abrogation of a specific DUSP gene does not produce a phenotypic change, the possibility of functional compensation by one of the other family members is difficult to exclude. To tackle this potential redundancy, cells or mice lacking more than one DUSP gene would be very helpful. The crossing of multiple DUSP-deficient mice to obtain combined knockouts is possible but very time-consuming and expensive. Hence, the application of CRISPR/Cas9 technology to target several DUSP genes of interest in a combinatorial manner appears very tempting and may help to overcome this technical hurdle.
- Comprehensive identification of the interaction partners and substrates of DUSP proteins
  Beyond the paradigm of classical MKP-DUSP functions as regulators of MAPK activity, evidence for the direct binding and dephosphorylation of other molecular interaction partners has been emerging. The massive advances in proteomics and phospho-proteomics methods in the last decade now provide the opportunity for an unbiased, in-depth investigation of DUSP interaction partners and to quantitatively assess the impact of DUSP overexpression or deletion on protein phosphorylation in cells. Such datasets can then also be employed for bioinformatics analysis of signaling pathways regulated by DUSP proteins. In addition, the increasing availability of structural information on DUSP proteins will facilitate the validation of candidate interactors by the computational modelling of their binding modes, an important prerequisite for the design and development of novel allosteric inhibitors of DUSPs. Depending on the specific DUSP, and on the cell type, such inhibitors may enhance immune responses and anti-microbial mechanisms, or conversely lead to the attenuation of inflammation. Therefore, the opposite approach to increase the expression or activity of certain DUSPs could be an attractive complementary or alternative strategy for the modulation of signaling and immune activation states.

## Abbreviations

CLP	Cecal ligation and puncture
DC	Dendritic cell
DUSP	Dual-specificity phosphatase
EAE	Experimental autoimmune encephalomyelitis
ICL	Idiopathic CD4 lymphopenia
ILC	Innate lymphoid cell
MAPK	Mitogen activated protein kinase
MKB	MAPK-binding domain
MKP	MAPK phosphatase
PRRPTP	Pattern recognition receptorProtein tyrosine phosphatase
TCR	T cell receptor
TLR	Toll-like receptor
